# Differentiation of extranodal non-Hodgkins lymphoma from squamous cell carcinoma of the maxillary sinus: a multimodality imaging approach

**DOI:** 10.1186/s40064-015-0974-y

**Published:** 2015-05-15

**Authors:** Hiroki Kato, Masayuki Kanematsu, Haruo Watanabe, Shimpei Kawaguchi, Keisuke Mizuta, Mitsuhiro Aoki

**Affiliations:** Department of Radiology, Gifu University School of Medicine, 1-1 Yanagido, Gifu, 501-1194 Japan; High-level Imaging Diagnosis Center, Gifu University Hospital, 1-1 Yanagido, Gifu, 501-1194 Japan; Department of Radiology, Gifu municipal Hospital, Gifu, Japan; Department of Otolaryngology, Gifu University School of Medicine, Gifu, Japan

**Keywords:** Maxillary sinus, Lymphoma, Squamous cell carcinoma, MRI, CT

## Abstract

This study aimed to assess the efficacy of a multimodality imaging approach for differentiating between primary extranodal non-Hodgkin’s lymphoma (NHL) and squamous cell carcinoma (SCC) of the maxillary sinus. Twelve NHLs and 29 SCCs of the maxillary sinus were included. CT findings, MR signal intensities, apparent diffusion coefficients (ADCs), and maximum standardized uptake values (SUVmax) were correlated with two pathologies. On CT, permeative growth frequency was greater among NHLs than among SCCs (50 % vs. 10 %; *p* < 0.01), whereas destructive growth frequency was greater among SCCs than among NHLs (83 % vs. 33 %; *p* < 0.01). On CT, remaining sinus wall within the tumor was more frequent with NHLs than with SCCs (92 % vs. 34 %; *p* < 0.01), whereas intratumoral necrosis was more frequent with SCCs than with NHLs (86 % vs. 17 %; *p* < 0.01). ADCs were lower for NHLs than for SCCs (0.61 vs. 0.95 × 10^–3^ mm^2^/s; *p* < 0.01). No significant differences in MR signal intensities and SUVmax were observed. Tumor growth pattern, remaining sinus wall within the tumor, and intratumoral necrosis were useful CT findings for differentiating between NHLs and SCCs. ADC measurements could assist the differentiation of NHL from SCC.

## Introduction

Malignant lymphoma is the most common non-epithelial head and neck malignancy. Although painless enlarged cervical lymph nodes comprise the most common presentation of lymphoma, the extranodal manifestations of lymphoma are well recognized and occur in 20–30 % of patients (Freeman et al. 1972). The head and neck is the second most common region for extranodal lymphoma after the gastrointestinal tract (Ezzat et al. 2001). Although the extranodal areas predisposed to lymphoma development, such as Waldeyer’s ring, are normally rich in lymphoid tissue (DePena et al. 1990), extranodal head and neck lymphoma also occur in the paranasal sinuses, nasal cavity, larynx, oral cavity, salivary glands, thyroid, and orbits.

Non-Hodgkin’s lymphoma (NHL) is the second most common malignancy in the paranasal sinuses and nasal cavity (Harbo et al. 1997). NHL involving the paranasal sinuses is frequently B-cell lymphoma (most commonly diffuse large B-cell lymphoma) (Abbondanzo and Wenig 1995; Nakamura et al. 1997). The early diagnosis of primary sinonasal NHL is difficult because this lesion develops in an anatomic space and expands toward the sinus, nasal cavity, or nasopharynx and does not usually cause symptoms in the early stages (Chalastras et al. 2007). The most common presenting symptoms of sinonasal NHL are nasal obstruction, epistaxis, headache, and unilateral facial, cheek, or nasal swelling (Chalastras et al. 2007). NHL is usually submucosal, and its gross appearance differs from that of squamous cell carcinoma (SCC), which is usually ulcerative.

It is important to differentiate sinonasal NHL from SCC because of the different treatment strategies. The patients with sinonasal NHL are usually treated with the combination of radiotherapy and chemotherapy. Meanwhile, surgical resection is the mainstay of treatment for the patients with sinonasal SCC, and radiotherapy or chemoradiotherapy are typically used in the adjuvant or neoadjuvant settings.

CT (Kondo et al. 1984; Nakamura et al. 1997; Yasumoto et al. 2000) and MR (Yasumoto et al. 2000) imaging findings of sinonasal NHL have been reported, and the differentiation of NHL from SCC of the maxillary sinus via CT findings has been discussed in light of the characteristics of primary tumors and cervical lymphadenopathy (Matsumoto et al. 1992; Urquhart et al. 2002). Furthermore, diffusion-weighted (DW) imaging is well known to be useful for differentiating NHL from SCC (Maeda et al. 2005; Abdel Razek et al. 2006; Sumi et al. 2007; Holzapfel et al. 2009). Recently, multimodality radiologic imaging is routinely performed for pretherapeutic assessment of the maxillary sinus malignancies. However, our literature search did not unearth any reports in which NHL was compared with SCC of the maxillary sinus via multiple imaging modalities including DW imaging and ^18^ F–fluorodeoxyglucose (FDG) PET/CT. Therefore, the purpose of this study was to assess the efficacy of a multimodality imaging approach for differentiating between primary NHL and SCC of the maxillary sinus.

## Materials and methods

### Patients

The study was approved by the human research committee of our institutional review board, and complied with the guidelines of the Health Insurance Portability and Accountability Act. The requirement for informed consent was waived due to the retrospective nature of this study. We examined our hospital’s electronic medical chart system for patients with histopathologically-proven primary maxillary sinus tumors between March 2005 and April 2014, and found 12 consecutive patients with NHL (age range, 46–83 years; mean age 65.6 years; 9 men and 3 women; diffuse large B-cell lymphoma in 12) and 29 with SCC (age range, 38–79 years; mean age 60.6 years; 23 men and 6 women; well-, moderately, and poorly differentiated SCC in 14, 6, and 9 patients, respectively). Clinical stages were stage I in 4 NHLs, stage II in 4 NHLs and 2 SCCs, stage III in 1 NHL and 10 SCCs, and stage IV in 3 NHLs and 17 SCCs. Pathologically or clinically proven tumor involvement of cervical nodes was found in 5 (42 %) of 12 NHLs and 6 (21 %) of 29 SCCs. CT was performed for all patients, MR imaging including DW imaging for 4 NHLs and 17 SCCs, and ^18^ F- FDG PET/CT for 7 NHLs and 15 SCCs. These radiological examinations were performed within 1 month for each individual patient. The patients’ characteristics are summarized in Table 1. The maximum diameter of NHLs ranged from 36 to 60 cm (mean 47.8 mm) and that of SCCs ranged from 32 to 80 cm (mean 49.4 mm).

**Table 1:** Patient characteristics

Characteristics	NHL	SCC
Number of patients	12	29
Age		
Mean	65.6	60.6
Range	46–83	38–79
Gender		
Male	9	23
Female	3	6
Clinical stage		
I	4	0
II	4	2
III	1	10
IV	3	17
Histological subtype		
B-cell lymphoma		
Diffuse large B cell lymphoma	12	
Well differentiated SCC		14
Moderately differentiated SCC		6
Poorly differentiated SCC		9
Examination		
CT	12	29
MRI	4	17
^18^ F- FDG PET/CT	7	15

Note.-- NHL = non-Hodgkin lymphoma, SCC = squamous cell carcinoma

### CT Imaging

An 8–slice CT scanner (LightSpeed Ultra; GE Healthcare, Milwaukee, WI, USA) was used for 32 patients (10 NHLs and 22 SCCs) and a 16–slice CT scanner (LightSpeed 16; GE Healthcare, Milwaukee, WI, USA) was used for remaining 9 patients (2 NHLs and 7 SCCs). Unenhanced CT images were obtained for all 41 patients (12 NHLs and 29 SCCs), and contrast-enhanced CT images were obtained for 30 patients (7 NHLs and 23 SCCs). Transverse CT images were reconstructed with 2.5–mm section thickness and no overlap. Coronal multiplanar reconstruction images with 2.5–mm section thickness were also obtained. These unenhanced CT images were reconstructed by using bone and soft-tissue algorithms. Contrast-enhanced CT images were obtained 45 s after initiating IV bolus injection of 100 mL of nonionic iodine contrast material containing 300–mg iodine per mL (Omnipaque300, Daiichi Sankyo, Tokyo, Japan) at an injection rate of 2 mL per second.

### MR Imaging

A 1.5–T MR imaging system (Intera Achieva 1.5 T Pulsar; Philips Medical Systems, Amsterdam, The Netherlands) was used. Unenhanced MR images were obtained for 21 patients (4 NHLs and 17 SCCs), and gadolinium-enhanced MR images were obtained for 16 patients (2 NHLs and 14 SCCs). Transverse MR images were obtained using the parallel imaging technique at 4–mm section thickness with 1–mm intersection gap. In 21 patients, non-fat suppressed T1-weighted spin-echo (TR/TE, 633–827/9–15 msec; imaging matrices, 512 × 512; field of view, 20 × 20 cm; parallel imaging factor, 1.5), non-fat suppressed T2–weighted fast spin-echo (TR/TE, 4000–5710/90–100 msec; imaging matrices, 512 × 512; field of view, 20 × 20 cm; parallel imaging factor, 1.5), and short-tau inversion recovery (STIR) single-shot spin-echo echo-planar DW (TR/TE/TI, 5490/72/170 msec; imaging matrices, 256 × 256; field of view, 40 × 40 cm; b-value, 0 and 1000 s/mm^2^; parallel imaging factor, 1.8) images were obtained. In 16 patients, gadolinium-enhanced fat-suppressed T1-weighted spin-echo images (TR/TE, 630–840/9–15 msec; imaging matrices, 512 × 512; field of view, 20 × 20 cm; parallel imaging factor, 1.5) were obtained after the intravenous injection of 0.1 mmol/kg of gadopentetate dimeglumine (Magnevist; Bayer Healthcare, Berlin, Germany).

### FDG-PET/CT

Whole-body PET/CT (Biograph Sensation 16; Siemens Medical Solutions, Malvern, PA, USA) from the skull to mid-thigh was performed for 22 patients (7 NHLs and 15 SCCs). Briefly, after at least 4 h of fasting, patients received an intravenous injection of ^18^ F-FDG (185 MBq). Blood glucose levels were checked in all patients before FDG injection, and no patient had a blood glucose level greater than 150 mg/dL. Approximately 60 min after FDG injection, CT and subsequent whole-body PET were performed.

Technical parameters of the 16–row multidetector CT were; a gantry rotation speed of 0.5 s, a table speed of 24 mm per a gantry rotation, and quiet-breathing data acquisition. Transverse images were reconstructed with 2 mm section thickness and no overlap. Oral or intravenous contrast agent was not used for CT. PET had an axial view of 16.2 cm per bed position with an intersectional gap of 3.75 mm in one bed position, which necessitated data acquisition in six or seven bed positions. Axial PET images were obtained using an imaging matrix of 256 × 256 and a field of view of 50 × 50 cm.

### Image assessment

Two radiologists with 15 and 9 years of post-training experience of head and neck imaging independently reviewed all of the CT images and evaluated the predominant tumor growth patterns. A permeative-type tumor was defined as an invasive lesion, which crossed the sinus wall accompanied by remaining original form of sinus walls as a linear structure within the tumor; a destructive-type tumor was defined as an invasive lesion accompanied by extensive bone destruction and no bony expansion of the adjacent maxillary sinus walls. An expansile-type tumor was defined as a non-invasive lesion accompanied by bony expansion or erosion of the adjacent maxillary sinus walls. The observers were unaware of the patients’ names, laboratory results, other imaging findings, or final diagnoses. Any disagreements between the reviewers were resolved through consensus.

The radiologist with 15 years of post-training experience of head and neck imaging also reviewed all of the CT images for the presence of remaining sinus wall within the tumor, intratumoral necrosis, cervical lymphadenopathy, and tumor extension. Permeative-type tumors were always accompanied by considerable amount of remaining sinus walls within the tumor. Among destructive and expansile-type tumors, if a little bit of sinus wall were found within the tumor, we assessed the presence of remaining sinus wall within the tumor. Among the cases with remaining sinus wall within the tumor, predominant bony erosive patterns were classified into resorption or remodeling, and the presence of accompanying sclerosis was also assessed. We regarded focal hypodense areas on unenhanced CT images or focal unenhanced areas on contrast-enhanced CT images as areas thought to be intratumoral necrosis. Enlarged cervical lymph nodes were considered as metastatic nodes when the minimum diameter exceeded 1.0 cm (van den Brekel et al. 1990). Cervical lymph nodes accompanied by focal hypodense areas on unenhanded CT image or focal unenhanced areas on contrast-enhanced CT images were also considered as metastatic nodes. Perineural spread was defined as an invasion of the pterygopalatine fossa, followed by extension to the greater or lesser palatine foramen, Vidian canal, foramen rotundum, or cavernous sinus. We used the following diagnostic criteria for perineural spread: asymmetric enlargement or destruction of the neural foramen on CT images.

In addition, the same reviewer defined the regions of interest (ROIs) in all of the MR sequences and recorded the MR signal intensities. ROIs were placed as broadly as possible in the tumors as widely as possible while excluding areas of necrosis. The reviewer also measured signal intensities of the spinal cord or brain stem at the same level as the tumors and calculated the tumor-to-spinal cord/brain stem signal intensity ratios. Apparent diffusion coefficient (ADC) values [×10^−3^ mm^2^/s] were measured on ADC maps by placing ROIs over the tumors. ROIs were placed to encompass lesions as much as possible while avoiding necrosis by referring to the T2–weighted images. For all ROI placements, the size, shape, and position of the ROIs were kept constant among the all sequences by applying a copy-and-paste function at the workstation.

For a semi-quantitative analysis of FDG uptake, the reviewer determined the maximum standardized uptake value (SUVmax) of each lesion.

### Statistical analysis

All statistical analyses were performed using SPSS version 18.0 (SPSS Inc., Chicago, IL, USA). The chi-square test or Fisher exact test was performed to compare the frequencies of imaging findings (predominant growth patterns, remaining sinus wall within the tumor, intratumoral necrosis, cervical lymphadenopathy, and tumor extension). The unpaired *t*-test was used to compare the quantitative results (tumor-to-spinal cord signal intensity ratios, ADC values, and SUVmax).

## Results

**Fig. 1:**
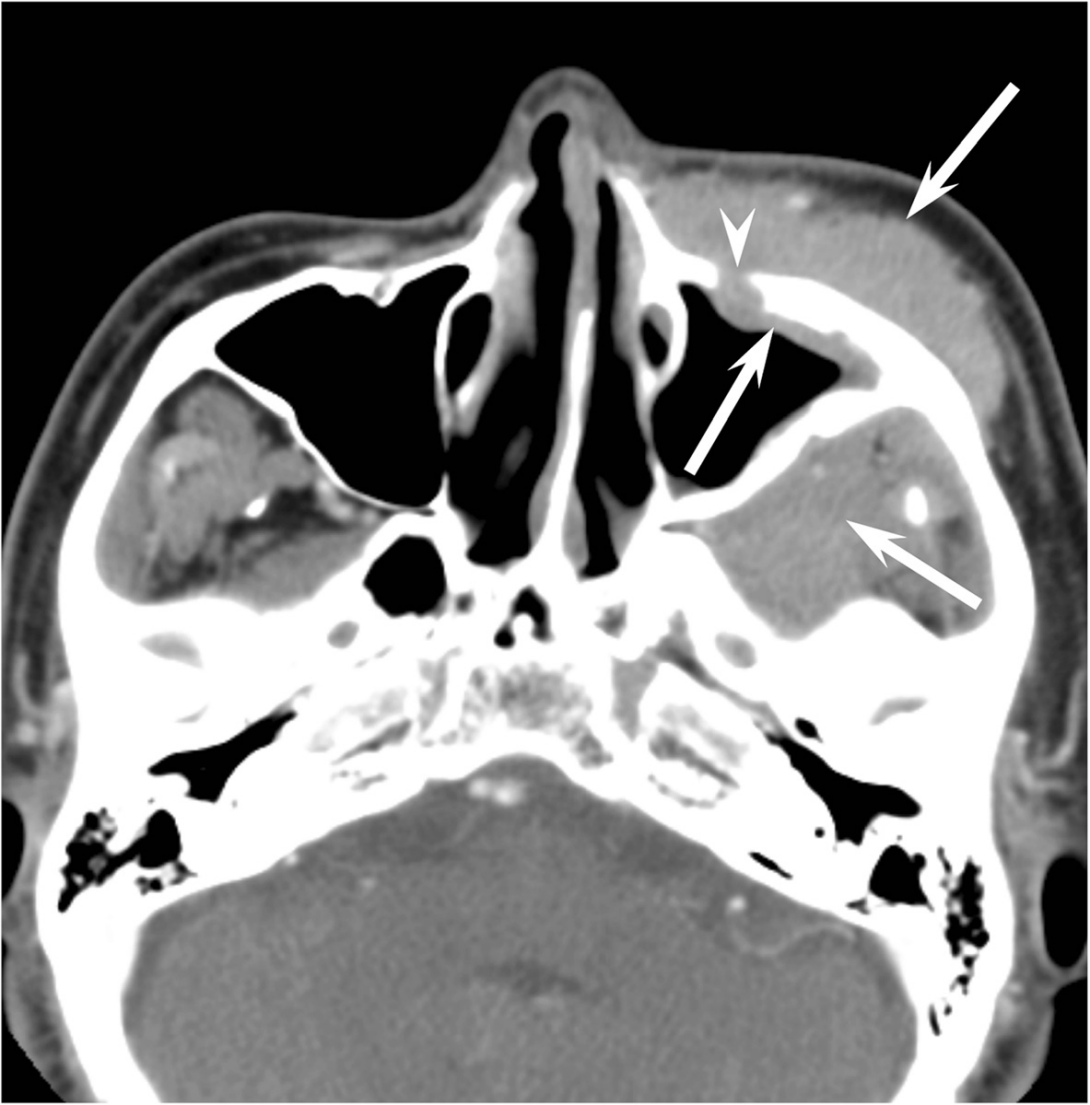
A 50–year-old man with maxillary sinus diffuse large cell lymphoma (permeative-type). Enhanced CT image shows a homogeneously enhanced mass inside and outside left maxillary sinus (arrow). The sinus walls are preserved due to the permeative growth, but infraorbital foramen (arrowhead) is slightly expanding

**Fig. 2:**
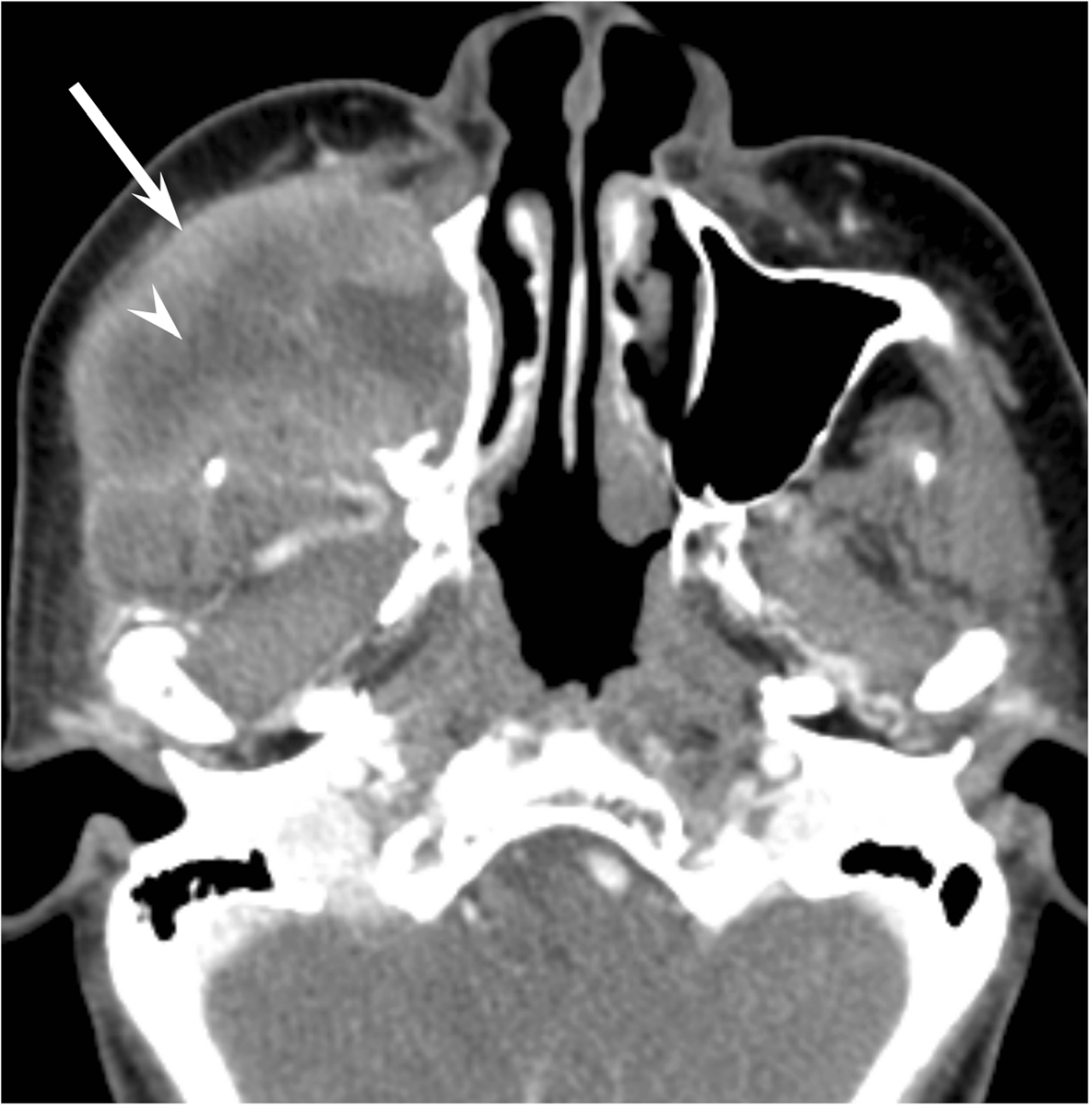
A 60–year-old woman with maxillary sinus squamous cell carcinoma (destructive-type). Enhanced CT image shows heterogeneously enhanced bulky mass (arrow) with unenhanced area suggestive of necrosis (arrowhead). The sinus walls extensively disappear due to the destructive growth

**Fig. 3:**
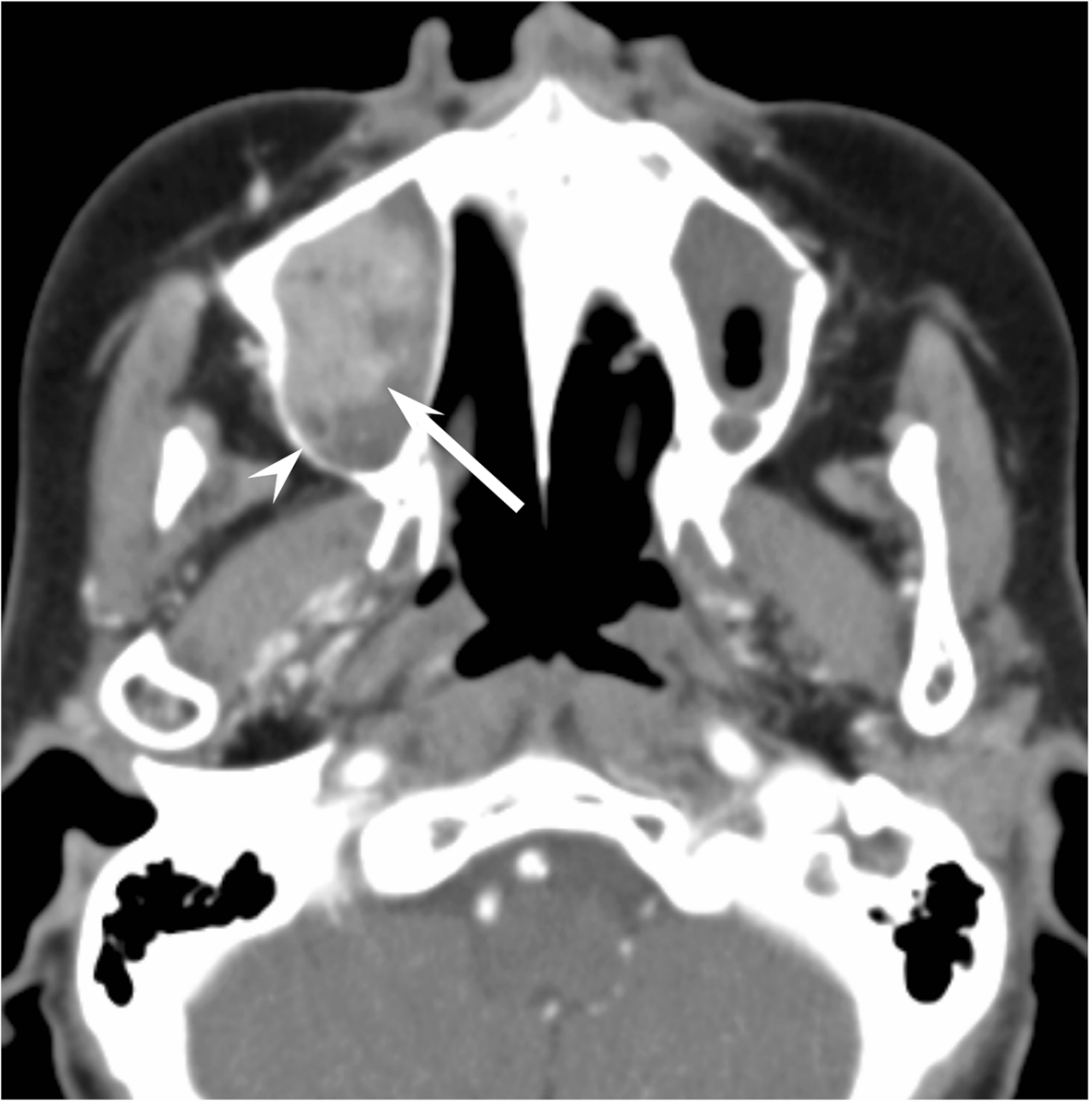
A 73–year-old woman with maxillary sinus squamous cell carcinoma (expansile-type). Enhanced CT image shows a heterogeneously enhanced mass of right maxillary sinus (arrow). The expansion and erosion of right posterior sinus walls is observed without bony defect (arrowhead)

The frequencies of the qualitative imaging findings and quantitative measurements in patients with NHL or SCC are summarized in Table 2. The predominant growth patterns were the permeative-type (Fig. 1) in 6/12 (50 %) and 3/29 (10 %), destructive-type (Fig. 2) in 4/12 (33 %) and 24/29 (83 %), and expansile-type (Fig. 3) in 2/12 (17 %) and 2/29 (7 %) of the NHLs and SCCs, respectively. Permeative growth was significantly more frequent among NHLs than among SCCs (*p* < 0.01), whereas destructive growth was significantly more frequent among SCCs than among NHLs (*p* < 0.01).

**Table 2:** Qualitative imaging findings and quantitative measurements with NHL and SCC of maxillary sinus

	NHL	SCC	*p* value
Qualitative Imaging Findings on CT	(n = 12)	(n = 29)	
Predominant growth pattern			
Permeative	6 (50)^a^	3 (10)	
Destructive	4 (33)	24 (83)^aa^	
Expansile	2 (17)	2 (7)	
Remaining sinus wall within the tumor	11 (92)^a^	10 (34)	0.001
Intratumoral necrosis	2 (17)	25 (86)^aa^	< 0.001
Cervical lymphadenopathy	4 (33)	4 (14)	0.158
Tumor extension			
Nasal cavity	9 (75)	22 (76)	0.622
Orbit	6 (50)	19 (66)	0.281
Subcutaneous tissue	9 (75)	17 (59)	0.266
Retroantral fat pad	10 (83)	24 (83)	0.672
Pterygoid process	2 (17)	10 (34)	0.226
Perineural spread	1 (8)	6 (21)	0.323
Intracranial	1 (8)	3 (10)	0.668
Quantitative Measurements on MRI	(n = 4)	(n = 17)	
T1–weighted images	0.89 ± 0.09	0.96 ± 0.18	0.457
T2–weighted images	1.10 ± 0.21	1.07 ± 0.25	0.813
Diffusion-weighted images	1.64 ± 0.68	1.13 ± 0.31	0.227
ADC (×10^−3^ mm^2^/s)	0.61 ± 0.09^aaa^	0.95 ± 0.12	< 0.001
Quantitative Measurements on PET/CT	(n = 7)	(n = 15)	
SUVmax	20.8 ± 7.4	17.4 ± 5.4	0.517

Note.-- NHL = non-Hodgkin lymphoma, SCC = squamous cell carcinoma. In qualitative imaging findings, data are numbers of patients, and numbers in parentheses are frequencies expressed as percentages. In quantitative measurements, deta are signal intensity ratio, ADC value, and SUVmax, with the mean ± 1 standard deviation

^a^The frequency of NHL was significantly greater than that of SCC (*p* < 0.01)

^aa^The frequency of SCC was significantly greater than that of NHL (*p* < 0.01)

^aaa^The value of NHL was significantly lower than that of SCC (*p* < 0.01)

Remaining sinus wall within the tumor was more frequently observed in NHLs than in SCCs (92 % vs. 34 %; *p* < 0.01), whereas intratumoral necrosis was more frequently observed in SCCs than in NHLs (86 % vs. 17 %; *p* < 0.01). Among the tumors with remaining sinus wall within the tumor, predominant bony erosive patterns were the bony resorption in 7/11 (64 %) and 8/10 (80 %) and the bony remodeling in 4/11 (36 %) and 2/10 (20 %) of the NHLs and SCCs, respectively. The accompanying sclerosis was observed in four (36 %) of 11 NHLs and in two (20 %) of 10 SCCs. No significant difference was found between NHLs and SCCs in terms of cervical lymphadenopathy (33 % vs. 14 %; *p* = 0.158) and tumor extension to the nasal cavity (75 % vs. 76 %; *p* = 0.622), orbit (50 % vs. 66 %; *p* = 0.281), subcutaneous tissue (75 % vs. 59 %; *p* = 0.266), retroantral fat pad (83 % vs. 83 %; *p* = 0.672), pterygoid process (17 % vs. 34 %; *p* = 0.226), perineural spread (8 % vs. 21 %; *p* = 0.323), and intracranial (8 % vs. 10 %; *p* = 0.668), respectively.

**Fig. 4:**
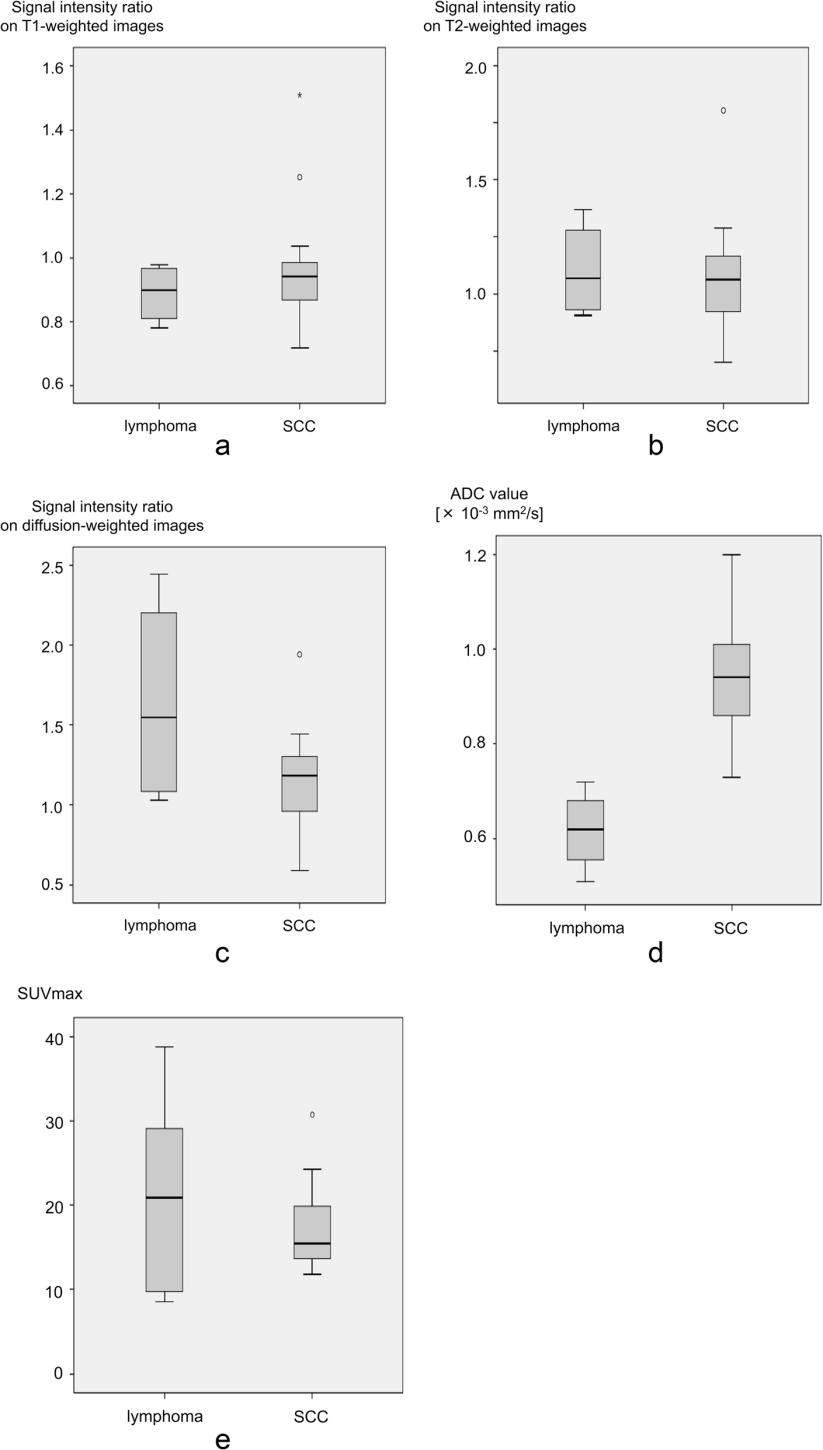
Box and whisker plots showing MR signal intensity ratios, ADC values, and SUVmax in patients with NHLs and SCCs. Boundary of boxes closest to zero indicates 25th percentile, line within boxes indicates median, and boundary of boxes farthest from zero indicates 75th percentile. Error bars indicate smallest and largest values within 1.5 box lengths of 25th and 75th percentiles. **a** No significant difference in signal intensity ratios on T1–weighted images was found between NHLs (0.89 ± 0.09) and SCCs (0.96 ± 0.18) (*p* = 0.457). **b** No significant difference in signal intensity ratios on T2–weighted images was found between NHLs (1.10 ± 0.21) and SCCs (1.07 ± 0.25) (*p* = 0.813). **c** No significant difference in signal intensity ratios on diffusion-weighted images was found between NHLs (1.64 ± 0.68) and SCCs (1.13 ± 0.31) (*p* = 0.227). **d** ADC value was significantly lower in NHLs (0.61 ± 0.09 × 10^−3^ mm^2^/s) than in SCCs (0.95 ± 0.12 × 10^−3^ mm^2^/s) (*p* < 0.01). **e** No significant difference in signal in SUVmax was found between NHLs (20.8 ± 7.4) and SCCs (17.4 ± 5.4) (*p* = 0.517)

The ADC value was lower among NHLs than among SCCs (0.61 ± 0.09 vs. 0.95 ± 0.12; *p* < 0.01) (Fig. 4). However, no significant differences were observed between NHLs and SCCs in terms of MR signal intensity ratios on T1–weighted images (0.89 ± 0.09 vs. 0.96 ± 0.18; *p* = 0.457), T2–weighted images (1.10 ± 0.21 vs. 1.07 ± 0.25; *p* = 0.813), and DW images (1.64 ± 0.68 vs. 1.13 ± 0.31; *p* = 0.227) and SUVmax (20.8 ± 7.4 vs. 17.4 ± 5.4; *p* = 0.517), respectively. The solid components of both NHLs and SCCs usually exhibited iso- to slightly low signal intensities on T1–weighted images and iso- to slightly high signal intensities on T2–weighted images compared with spinal cord or brain stem.

## Discussion

Although sinonasal lymphomas frequently shows infiltrative or permeative bony invasion, they cause various degrees of regional bone destruction. However, the bony resorption or remodeling caused by lymphoma may be accompanied by bone sclerosis as well (DePena et al. 1990; Matsumoto et al. 1992). Therefore, radiologists should know that various types of neighboring bony changes can occur with sinonasal lymphoma. On the other hand, maxillary sinus SCCs usually showed aggressive bony destruction in the adjacent sinus walls (Matsumoto et al. 1992). Our results revealed that permeative growth was more frequently observed with NHLs than with SCCs, whereas destructive growth was more frequently observed with SCCs than with NHLs. Because the frequency of permeative growth of SCCs was low, NHLs should be considered when CT images demonstrate maxillary sinus tumors with predominantly permeative growth.

Because untreated NHLs are not accompanied by calcification, calcification within the maxillary sinus NHL may well indicate bony sinus walls fragments. Therefore, calcification within the maxillary sinus NHLs usually has a thin and often linear appearance (Yasumoto et al. 2000). In our series, remaining sinus wall within the tumor was more frequently observed with NHLs than with SCCs. Although our results showed that remaining sinus wall was found in one third of maxillary sinus SCCs, NHLs should be considered when most of sinus walls are preserved for the extensive tumor involvement.

Because hypoxia is a common feature in most cases of SCC, prolonged oxygen deprivation will lead to chronic hypoxic stress and consequent tumor necrosis (Li et al. 2012). Central necrosis is one of the characteristic findings of metastatic cervical nodes from SCCs, and the detection of nodal necrosis in patients with primary head and neck SCCs is the most reliable sign of metastatic nodes. Necrosis is more frequently observed in the primary tumors of maxillary sinus SCCs than in those of NHLs in the previous literature (Urquhart et al. 2002). However, bulky NHLs of the maxillary sinus could be accompanied by intratumoral necrosis.

Nodal involvement is found in half of all patients with extranodal NHL of the head and neck either at presentation or during the course of disease (Hanna et al. 1997; King et al. 2004). Nodal disease is particularly associated with NHL of Waldeyer’s ring and NHL of the tonsil in particular, which tends to present as B-cell lymphoma (Yuen and Jacobs 1999). Although the imaging findings of metastatic lymph nodes from SCCs are usually characterized by central necrosis and ring enhancement, no significant differences have been observed between maxillary sinus NHLs and SCCs in the total number of nodes, number of large nodes, or maximum nodal diameter (Urquhart et al. 2002). However, Posterior triangle node, upper visceral nodes, and superior mediastinal node involvement have been reported to occur more frequently with the maxillary sinus NHL than with SCC (Urquhart et al. 2002). Although the frequency of pathologically or clinically proven tumor involvement of cervical nodes tended to be higher with NHLs than with SCCs, our CT criteria of cervical lymphadenopahy seemed to underestimate cervical nodal involvement.

As ADCs on DW imaging have been reported to correlate strongly with cellularity (Guo et al. 2002), the ADCs of lymphomas usually appear smaller than those of SCC (Maeda et al. 2005; Abdel Razek et al. 2006; Sumi et al. 2007; Holzapfel et al. 2009). In the sinonasal region, ADCs of each sinonasal pathology have not been sufficiently assessed, although it has been reported that the mean ADCs of malignant sinonasal tumors are significantly lower than those of benign sinonasal lesions (White et al. 2006; Razek et al. 2009; Sasaki et al. 2011). Because our results revealed that DW imaging with ADC measurements could effectively differentiate NHL from SCC of the maxillary sinus, further research progress regarding the DW imaging of other sinonasal pathologies is expected.

Although FDG-PET has been demonstrated to be useful for the initial diagnosis, staging, recurrence detection, and chemo- and radio-therapeutic response determination in head and neck malignancies (Gordin et al. 2007; Al-Ibraheem et al. 2009), the usefulness of FDG-PET for differentiating NHL from SCC in the head and neck region has not been yet established.

The differential diagnosis of maxillary sinus NHLs and SCCs include many other histological types. Because of permeative growth were frequency observed in undifferentiated neoplasms, the representative differential diagnosis of maxillary sinus NHLs includes so-called small round cell tumors. Small round cell tumors of the maxillary sinus include sinonasal undifferentiated carcinoma, small cell carcinoma (neuroendocrine type), extraosseous Ewing sarcoma/primitive neuroectodermal tumor (PNET), and extramedullary plasmacytoma (Iezzoni and Mills 2005). Meanwhile, because high-grade malignancy except small round cell tumors usually demonstrates destructive growth, the representative differential diagnosis of maxillary sinus SCCs includes high-grade carcinoma and sarcoma.

The present study has several limitations. First, the study sample was small because the study was conducted at a single institution. Second, given the retrospective nature of this study, ADCs and SUVmax were not available for the entire study population because both MR imaging and PET/CT were not performed for all patients. Third, we did not perform contrast-enhanced CT or MR imaging for all patients; and thus, the prevalence of intratumoral necrosis and tumor extension may not have been accurately assessed. These limitations of small samples and unstandardized imaging examinations should have been previously considered and can influence the results significantly.

## Conclusion

In summary, permeative growth, remaining sinus wall within the tumor, and the absence of intratumoral necrosis were more frequently observed with NHLs than with SCCs of the maxillary sinus. NHLs with permeative growth were always found to contain bony fragments of the remaining sinus wall. Although no significant differences in the MR signal intensity ratios and SUVmax were observed between NHLs and SCCs, DW imaging with ADC measurements could assist with the differentiation of NHLs from SCCs of the maxillary sinus.

## Abbreviations

NHL: Non-Hodgkin’s lymphoma
SCC: Squamous cell carcinoma
ADC: Apparent diffusion coefficient
SUVmax: Maximum standardized uptake value
DWI: Diffusion-weighted imaging
FDG: ^18^ F-fluorodeoxyglucose
STIR: Short-tau inversion recovery
ROI: Regions of interest
